# Complete Genome Sequence of *Bradyrhizobium* sp. Strain C-145, a Nitrogen-Fixing Rhizobacterium Used as a Peanut Inoculant in Argentina

**DOI:** 10.1128/mra.00505-22

**Published:** 2022-07-19

**Authors:** Fiorela Nievas, Santiago Revale, Emiliano Foresto, Sacha Cossovich, Mariana Puente, Pedro Alzari, Mariano Martínez, Mathilde Ben-Assaya, Damien Mornico, Maricel Santoro, Francisco Martínez-Abarca, Walter Giordano, Pablo Bogino

**Affiliations:** a Instituto de Biotecnología Ambiental y Salud (INBIAS-CONICET), Departamento de Biología Molecular, Facultad de Ciencias Exactas, Físico-Químicas y Naturales, Universidad Nacional de Río Cuarto, Río Cuarto, Córdoba, Argentina; b Wellcome Centre for Human Genetics, University of Oxford, Oxford, United Kingdom; c Instituto Nacional de Tecnología Agropecuaria (INTA), Instituto de Microbiología y Zoología Agrícola, Castelar, Argentina; d Unité de Microbiologie Structurale, Institut Pasteur, CNRS UMR 3528, Université de Paris, Paris, France; e Hub de Bioinformatique et Biostatistique—Département Biologie Computationnelle, CNRS USR 3756, Institut Pasteur, Paris, France; f Department of Biochemistry, Max Planck for Chemical Ecology, Jena, Germany; g Structure, Dynamics, and Function of Rhizobacterial Genomes, Department of Plant and Soil Microbiology, Estación Experimental del Zaidín—CSIC, Granada, Spain; University of Arizona

## Abstract

We present the complete genome sequence of *Bradyrhizobium* sp. strain C-145, one of the most widely used nitrogen-fixing rhizobacteria for inoculating peanut crops in Argentina. The genome consists of 9.53 Mbp in a single circular chromosome and was determined using a hybrid long- and short-read assembly approach.

## ANNOUNCEMENT

The symbiosis between legumes and bacteria from the *Bradyrhizobium* genus is one of the most efficient and economically important worldwide (1). Inoculation of soybean and peanut crops with effective bradyrhizobia is an environmentally friendly alternative to chemical fertilization (2–4). The peanut-nodulating strain *Bradyrhizobium* sp. strain TAL 1371 (NifTAL code) was acquired by the Instituto Nacional de Tecnología Agropecuaria (INTA; Argentina) from the University of Texas (5). After being evaluated and reisolated from peanut cultivars, it was renamed *Bradyrhizobium* sp. strain C-145 and became the recommended choice for peanut inoculation. In symbiosis with this legume, it outperforms other *Bradyrhizobium* sp. strains in terms of nitrogen-fixing ability, productivity, and environmental competitiveness (4, 6–8). This is particularly relevant given the prominence of peanut cultivation in central Argentina (9). Knowledge of the strain’s genome is crucial for maintaining and developing further the current agricultural model.

To date, although 581 genome assemblies are registered in NCBI for *Bradyrhizobium* strains, most are draft sequences. No genome data were available until now for strains used in commercial peanut inoculants.

Here, we introduce the complete genome sequence of *Bradyrhizobium* sp. C-145. A pure culture of the strain, provided by INTA, was grown in liquid yeast extract-mannitol medium (5). This was the source for the total DNA, obtained using a DNeasy blood and tissue kit (Qiagen) for Illumina sequencing and using a Promega Wizard high-molecular-weight (HMW) DNA extraction kit (Promega) for Oxford Nanopore Technologies sequencing. Illumina sequencing was performed on the P2M (Plateforme de Microbiologie Mutualisée) platform at Institut Pasteur. The library was prepared using a Nextera XT DNA library preparation kit and then sequenced on an Illumina NextSeq 500 instrument in paired-end (PE) 150-bp read configuration. Nanopore sequencing was carried out at the Oxford Genomics Centre. The sample was processed using both an Oxford Nanopore Technologies rapid barcoding sequencing kit (SQK-RBK004) and a native barcoding genomic DNA sequencing kit (SQK-LSK109 with EXP-NBD104). The products of each were sequenced in two Flongle flow cells. Data were base called using Guppy v4.2.2 using high-accuracy mode and the –trim_barcodes option. We obtained 6,542,626 Illumina PE reads and 41,545 Nanopore long reads (average, 5,030 bp), predicting 102-fold and 22-fold coverage, respectively. Hybrid genome assembly was performed on the raw reads using the nf-core/bacass pipeline (commit ceebac0) with default parameters (10). The assembly resulted in one contig that was closed by manually analyzing the overlapping ends using Geneious Basic (11). Accordingly, the assembly revealed a single chromosome of 9,529,571 bp with 62.9% G+C content, in line with what is known about the genus *Bradyrhizobium* (12). The genome, annotated using the NCBI Prokaryotic Genomes Automatic Annotation Pipeline (PGAAP) (13–15), consists of 8,500 protein-coding sequences, a single ribosomal operon, and 49 tRNAs. Like other *Bradyrhizobium* strains (16), C-145 features symbiotic islands in two zones (coordinates 1250 to 1535 kb and 6370 to 6750 kb), with a low GC content (59.8 and 58.5%, respectively), including most of the *nod*, *nif*, and *fix* genes. There are also several genes associated with the rhizospheric lifestyle (motility, exopolysaccharide production) and type I, II, III, and IV secretion systems.

This complete genome of a strain extensively used for peanut inoculation will enable more in-depth, comparative genomic analyses to elucidate the specific mechanisms behind *Bradyrhizobium*-peanut interactions.

### Data availability.

The complete genome sequence of *Bradyrhizobium* sp. C145 is available at NCBI GenBank under accession CP088150, BioProject accession number PRJNA782308, and BioSample accession number SAMN23371896. The raw data reads are available at NCBI’s Sequence Read Archive under accession numbers SRR17030678 to SRR17030682.
